# Changes in Surface Charge Density of Blood Cells in Fatal Accidental Hypothermia

**DOI:** 10.1007/s00232-015-9838-1

**Published:** 2015-09-12

**Authors:** Michał Szeremeta, Aneta Dorota Petelska, Joanna Kotyńska, Witold Pepiński, Monika Naumowicz, Zbigniew Artur Figaszewski, Anna Niemcunowicz-Janica

**Affiliations:** Department of Forensic Medicine, Medical University of Bialystok, Waszyngtona St. 13, 15-230 Bialystok, Poland; Institute of Chemistry, University of Bialystok, K. Ciolkowskiego St. 1K, 15-245 Bialystok, Poland; Laboratory of Electrochemical Power Sources, Faculty of Chemistry, University of Warsaw, Pasteur St. 1, 02-093 Warsaw, Poland

**Keywords:** Fatal accidental hypothermia, Surface charge density, pH measurement, Erythrocyte, Thrombocyte

## Abstract

The objective of this research was to evaluate postmortem changes concerning electric charge of human erythrocytes and thrombocytes in fatal accidental hypothermia. The surface charge density values were determined on the basis of the electrophoretic mobility measurements of the cells conducted at various pH values of electrolyte solution. The surface charge of erythrocyte membranes after fatal accidental hypothermia increased compared to the control group within whole range of experimental pH values. Moreover, a slight shift of the isoelectric point of erythrocyte membranes towards high pH values was observed. The surface charge of thrombocyte membranes in fatal accidental hypothermia decreased at low pH compared to the control group. However, at pH range 4–9, the values increased compared to the control group. The isoelectric point of thrombocyte membranes after fatal accidental hypothermia was slightly shifted towards low pH values compared to the control group. The observed changes are probably connected with the partial destruction and functional changes of the blood cell structure.

## Introduction

Hypothermia occurs when the core body temperature is 35 °C, and in such a situation, it is classified as mild. Within the range of 32–28 °C, hypothermia is defined as moderate and as severe at <28 °C (Mallet 2002). There are different ways of classifying hypothermia; the simplest one is to divide fatalities into those occurring in dry environment—on land and those occurring in water—that is in wet environment or immersion hypothermia (Palmiere et al. 2014a, b). Until the 50s of the twentieth century, there was a popular misconception that the death due to hypothermia concerns individuals living outdoors, in an environmental with temperature below 0 °C. However, according to the current state of knowledge, death caused by hypothermia may occur when the ambient temperature is between 0 °C and +10 °C. The degree of susceptibility to hypothermia is an individual matter.

Due to non-specific findings, the postmortem diagnosis of hypothermia is problematic. During the autopsy, macroscopic features of hypothermia usually include pink discoloration of the skin, especially around the elbows and knees (the so-called frost erythema), red lividity, red blood, hemorrhagic spots of the gastric mucosa (Wiszniewski’s spots), pancreatitis hemorrhages, synovial membrane hemorrhages, and hemorrhages into the large muscles of the body, especially into iliopsoas muscle (Tsokos et al. 2006; Bright et al. 2013).

On the other hand, pathophysiological changes associated with fatal accidental hypothermia are well described as effects in cardiovascular system, hematology, neuromuscular, and respiratory systems (Mallet 2002; Zhu et al. 2002, 2006a, b, 2007a, b, c). One can indicate gastrointestinal effects and renal and metabolic irregulations as well (Zhu et al. 2002, 2005; Quan et al. 2010). It is also possible to characterize the chemical and biochemical features of being exposed to cold. Postmortem biochemical examination revealed few issues connected to acid–base, electrolyte, fluid balance, catecholamines, and keton bodies in cases of fatal accidental hypothermia (Mant 1964, 1969, Hirvonen 1976; Hirvonen and Huttunen 1982; Zhu et al. 2007a, b; Li et al. 2009; Jakubeniene et al. 2009a, b; Ishikawa et al. 2010).

When it comes to coagulation system, it is a balanced combination of prothrombotic, anticoagulant, and fibrinolytic processes which is complex yet delicate. According to popular conception, hypothermia reduces coagulation and platelet function and impairs primary and secondary hemostasis. The hematological changes connected to hypothermia result from the increase in blood viscosity, fibrinogen, and hematocrit. These alternations can cause the disorders concerning the function of many organs. Alternations in vascular permeability cause the loss of plasma to extravascular compartments which leads to hemoconcentration. The hematocrit increases by about 2 % for every 1 °C decline in temperature (Danzl and Pozos 1994), and a normal hematocrit in a case of moderately or severely hypothermic individual indicates pre‐existing anemia or blood loss. It has also been indicated that hypothermia can cause marrow suppression and progressive marrow failure as well as introduction of erythroid hypoplasia and sideroblastic anemia (O’Brien et al. 1982; Rosenkranz 1985).

The direct influence of hypothermia concerns the enzymic reactions of both intrinsic and extrinsic pathways of the clotting cascade (Rohrer and Natale 1992), and, as a result, coagulopathy can occur. Prothrombin time, thrombin time, and partial thromboplastin time are affected by the low temperature. Appropriate management involves rewarming, rather than administration of exogenous clotting factors (Reed et al. 1992). On a number of occasions, a disseminated intravascular coagulopathy has been reported without any apparent cause other than the hypothermia itself (Breen et al. 1988); such a situation may result from the release of tissue thromboplastin from ischaemic tissue (Mahajan et al. 1981), or the circulatory collapse could turn out to be the major factor (Carden and Nowak 1982). Moreover, hypothermia can impair both the endothelial synthesis of prostacyclin (PGI_2_) and its inhibitory action connected to platelet aggregation, promoting platelet activation and thrombosis (Mikhailidis et al. 1983). What is more, platelet production of thromboxane B_2_ depends on the temperature, and it encourages a decline in platelet activity when the temperature decreases (Easterbrook and Davis 1985).

As there is no sufficient number of professional literatures about the influence of hypothermia on the electrical properties of biological membranes, we researched the changes of surface charge density of blood cells after fatal accidental hypothermia. It is a continuation of a systematic study of the electrical properties of postmortem human erythrocyte and thrombocyte membranes, which was examined by Figaszewski and co-workers (Kotyńska et al. 2012; Szeremeta et al. 2013; Petelska et al. 2015). The researchers conducted their experience using a microelectrophoresis method since it is one of the fundamental analytical tools in case of biological studies. The electrophoretic mobility measurements were carried out with a pH range of 2–11. From our perspective, the results we obtained can be helpful both in the interpretation as well as understanding of the processes which take place on biological membrane surfaces after fatal accidental hypothermia.

## Materials and Methods

Blood (pH 6.6) was obtained from sober individuals during autopsies conducted at the Forensic Medicine Department at the Medical University of Bialystok in the year 2013. The examination was based on 10 selective examples of fatal hypothermia (five men and five women, mean age 43.7 years, range 23–71) autopsied in the year 2013. Approval for this study was granted by the Ethics Review Board of the Medical University of Bialystok (No. R-I-002/533/2010).

Blood was obtained from all individuals during autopsies conducted at the Forensic Medicine Department at the Medical University of Bialystok in the year 2013. Blood was routinely obtained from the femoral vein, inserted into chemically and biologically clean glass containers, and donated to the Department of Electrochemistry at the University of Bialystok. The donated samples were analyzed comparatively with control samples taken from live individuals that are from healthy volunteers.

## Preparation of Erythrocytes from Blood

Erythrocytes were isolated from 2 ml of liquid whole blood by centrifugation at 900×*g* for 8 min at room temperature. The supernatant thrombocyte-rich plasma was removed and saved for subsequent processing, while the erythrocytes were washed three times with isotonic saline (0.9 % NaCl) at 3000×*g* for 15 min. After the final wash, the erythrocyte pellet was resuspended in isotonic saline for electrophoretic measurement.

## Preparation of Thrombocytes from Plasma

Thrombocyte-rich plasma was centrifuged at 4000×*g* for 8 min. The supernatant plasma was removed and discarded. The thrombocyte pellet was washed three times with isotonic saline by centrifugation at 3000×*g* for 15 min. After the final wash, thrombocytes were resuspended in isotonic saline for electrophoretic measurement.

All solutions and cleaning procedures were performed using water which was purified thanks to Milli-Qll system (18.2; Millipore, Billerica, MA).

## Microelectrophoretic Mobility Measurements

The electrophoretic mobility of erythrocyte or thrombocyte vesicles in suspension was measured using Doppler velocimetry laser and a Zetasizer Nano ZS (Malvern Instruments, Malvern, UK) apparatus. Measurements were carried out as a function of pH. Cell membranes were suspended in NaCl solution and titrated to the desired pH using HCl or NaOH. The reported values represent the average of at least six measurements performed at a given pH.

Based on electrophoretic mobility measurements, the surface charge density was determined using Eq. 1 (Alexander and Johnson 1949):

1 $$\delta = \frac{\eta \cdot u}{d}\, ,$$

where *η* is the viscosity of the solution, *u* is the electrophoretic mobility, and *d* is the diffuse layer thickness.

The diffuse layer thickness (Barrow 1996) was determined according to the formula:

2 $$d = \sqrt {\frac{{\varepsilon \cdot \varepsilon_{0} \cdot R \cdot T}}{{2 \cdot F^{2} \cdot I}}} \, ,$$

where *R* is the gas constant, *T* is temperature, *F* is the Faraday number, *I* is the ionic strength of 0.9 % NaCl, and *εε*_0_ is the permeability of the electric medium.

## Results and Discussion

The grounds for investigations on membrane association phenomena were constituted by electrophoretic mobility measurements conducted after fatal accidental hypothermia occurred in blood cells. The experiments were performed at several pH values using 0.155 M NaCl which served as a supporting electrolyte. Then the electrophoretic mobility values were converted to surface charge density. It was done by Eq. 1 presented in section Materials and Methods.

**Fig. 1:**
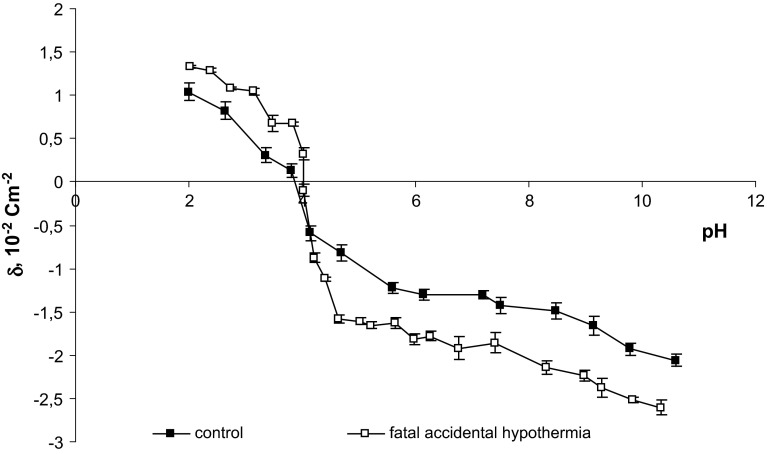
pH dependence of surface charge density of erythrocytes

The surface charge densities of erythrocytes of control and fatal accidental hypothermia are plotted as a function of pH in Fig. 1. The experiment indicated an increase in positive charge of the erythrocytes membrane after fatal accidental hypothermia when compared to control erythrocytes when acid solution was used. On the other hand, an increase in negative charge after fatal accidental hypothermia when compared to control erythrocytes was indicated in case of using basic solution.

Table 1 presents isoelectric point and surface charge density values for human erythrocytes. These data were obtained using electrophoresis method. The presentation of data has a form of mean standard deviation. The data were a subject of analysis conducted based on standard statistical analysis.

**Table 1:** The surface charge density and isoelectric point values for human erythrocytes (control and fatal accidental hypothermia)

Groups	Isoelectric point	Surface charge density [10^−2^ C m^−2^]	
		At low pH values	At high pH values
Control (Kotyńska et al. 2012; Szeremeta et al. 2013)	3.72	1.039 ± 0.103	−2.063 ± 0.059
Fatal accidental hypothermia	3.80	1.334 ± 0.117	−2.603 ± 0.091

Whereas fatal accidental hypothermia has specific and strong influence on the acid–base balance, the body temperature decreases opposite thing happens to solubility oxygen and carbon dioxide levels and the activity of histidine imidazole ring buffer included in the hemoglobin increases. According to estimations, in a closed system with every degree drop in temperature, the pH is increased by 0.015—Rosenthal formula (Rosenthal 1948). Thanks to that formula, one could probably provide explanation of the pH levels (~6.6) indicated in the blood samples we collected. Consequently, pH increases at an early stage of hypothermia and what usually decreases is the pressure of the carbon dioxide. In the following stages, one can observe the hyperkalemia associated with acidosis and renal failure (Mallet 2002). What is more, in case of accidental fatal hypothermia, alternations of surface charge density can be also connected to the production of ketone bodies in the liver as an alternative energy source and hormonal stimulation of hypothalamic-pituitary-adrenal axis. The latter also advances concentration of ketone bodies, mainly β—hydroxybutyric acid in sober individuals (Palmiere et al. 2014a; Bańka et al. 2014). However, if fatal accidental hypothermia occurs, the oxygen flow to tissues decreases which results in an energetic deficit that can compensate for only activation of anaerobic ATP supply pathway (Pasteur effect). Due to the rapid depletion of fermentable substrate together with the accumulation of deleterious end-products, like H^+^ ions (Boutilier 2001) anaerobic ATP production cannot sustain the pre-existing energy demands of cells, as well as tissues. Alternatively, differential permeabilities of the major ions themselves, with little or no direct involvement of the Na+/K+ ATPase (Plesnila et al. 2000), could be the cause of the membrane changes (destabilization) during accidental hypothermia.

**Fig. 2:**
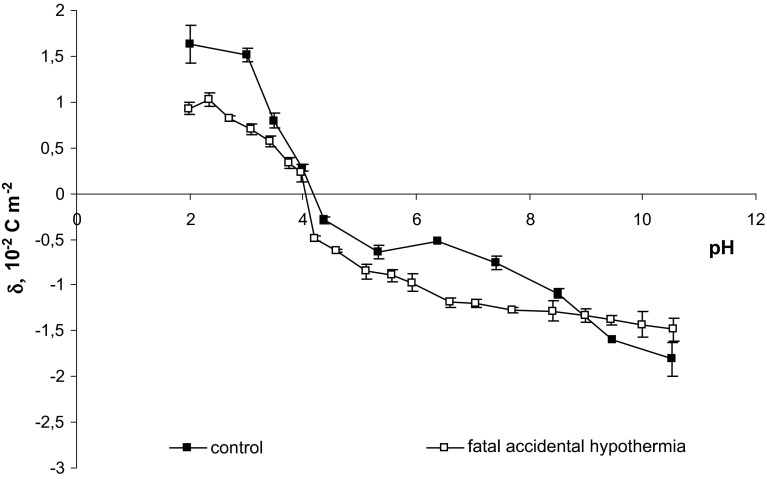
pH dependence of surface charge density of thrombocytes

The surface charge densities of thrombocytes of the control and after fatal accidental hypothermia are plotted as a function of pH in Fig. 2. The decrease in positive charge of the membrane in a pH range 2–4 when compared with control membrane is a result of hypothermia. Within pH range 4–9, hypothermia leads to an increase in negative charge of the thrombocytes membrane, and there is a little shift of the isoelectric point of the membrane towards low pH values. Table 2 contains isoelectric point and surface charge density values for human thrombocyte.

**Table 2:** The surface charge density and isoelectric point values for human thrombocytes (control and fatal accidental hypothermia)

Groups	Isoelectric point	Surface charge density [10^−2^ C m^−2^]	
		At low pH values	At high pH values
Control (Kotyńska et al. 2012; Szeremeta et al. 2013)	4.20	1.628 ± 0.206	−1.810 ± 0.185
Fatal accidental hypothermia	4.10	0.929 ± 0.071	−1.486 ± 0.128

In our view, such alternations may be, to much extent, dictated by the same processes as described in the case of erythrocytes. Quite the opposite, the differences observed in surface charge densities could have been cause by coagulopathy, and it manifested itself by a gradual impairment of platelet function (TXB_2_), thrombocytopenia, decrease in the activity of clotting factors, and increased fibrinolytic activity. However, one must bear in mind hypothermia can impair both the endothelial synthesis of prostacyclin (PGI_2_) and its inhibitory action on platelet aggregation, promoting platelet activation. In other words, platelets secrete granule products that include calcium (actives coagulation proteins) and ADP (mediates further platelets aggregations and degranulation). Phospholipids complexes are also exposed by activated platelets which are the source of an important surface for coagulation-protein activation (Kumar et al. 2007). However, at this stage, we are not able to declare which mechanism leads to the examined surface charge density of platelets in fatal accidental hypothermia in greater degree.
